# Beyond Nitrogen Fixation: Multifunctional Roles of Common Bean in Organic Agroecosystems and Ecosystem-Oriented Breeding Strategies

**DOI:** 10.3390/plants15182884

**Published:** 2026-09-21

**Authors:** Dan Ioan Avasiloaiei, Mariana Calara, Petre Marian Brezeanu, Barbara Pipan, Lovro Sinkovič, Creola Brezeanu

**Affiliations:** 1 Vegetable Research and Development Station, Calea Barladului Street, no. 220, 600388 Bacau, Romania; calaramariana@gmail.com (M.C.); brezeanumarian@legumebac.ro (P.M.B.); 2 Group for Plant Genetics and Breeding, Crop Science Department, Agricultural Institute of Slovenia, Hacquetova ulica 17, 1000 Ljubljana, Slovenia; barbara.pipan@kis.si (B.P.); lovro.sinkovic@kis.si (L.S.)

**Keywords:** holobiont, rhizosphere microbiome, ecosystem-oriented breeding, resource-use efficiency, ecological intensification

## Abstract

Common bean (*Phaseolus vulgaris* L.) is among the world’s most widely consumed grain legumes and a foundational species for organic and low-input agriculture, yet its agroecological value extends far beyond nitrogen fixation and nutritional provisioning. This review reframes *P. vulgaris* as a breeding target defined by ecosystem function rather than by yield alone, synthesizing evidence on functional biology, biological nitrogen fixation, rhizosphere microbiome assembly and soil ecosystem engineering to identify the trait complexes that ecosystem-oriented breeding programs must select for. We examine the constraints limiting expression of these traits under organic management, including weed competition, biotic and abiotic stress, nutrient limitation, and the scarcity of cultivars and certified seed adapted to low-input environments, and evaluate the agroecological management practices, resource-use efficiency metrics and emerging technologies—spanning precision agriculture, phenomics, molecular breeding, genome editing and microbiome engineering—that can accelerate their genetic improvement. Central to this synthesis is a paradigm shift in breeding objectives: common bean is conceptualized as a holobiont whose performance emerges from coordinated plant–microbiome interaction, requiring breeding programs to select explicitly for nitrogen-fixation efficiency, root system architecture, rhizosphere recruitment capacity, nutrient-use efficiency and multi-stress resilience as heritable, ecosystem-oriented traits. We conclude by outlining priority breeding research directions integrating genomics, phenomics, envirotyping and rhizosphere ecology, positioning common bean breeding as a strategic lever for the transition toward regenerative, climate-smart organic agriculture.

## 1. Introduction

Global agriculture faces the intertwined pressures of food insecurity, climate change, biodiversity loss and declining soil fertility, and the transition toward resilient agroecosystems increasingly depends on crops bred to deliver multiple ecological functions beyond primary productivity. Among grain legumes, common bean (*Phaseolus vulgaris* L.) has traditionally been valued for its nutritional importance and biological nitrogen fixation (BNF), yet its contribution to sustainable agriculture extends far beyond these attributes, encompassing rhizosphere microbiome engineering, nutrient cycling and above- and belowground biodiversity support [1,2,3,4]. *P. vulgaris* should therefore be regarded not merely as a legume crop but as a multifunctional agroecological species whose genetic improvement must be redefined accordingly.

This review therefore adopts a breeding-oriented, mechanism-driven perspective, asking not how organic beans are cultivated, but which biological functions the species performs; which constraints limit expression of these functions; and which traits, tools and selection strategies can translate them into ecosystem-oriented cultivars. Because climate variability increasingly manifests as combined and recurrent—rather than isolated, stress events—climate resilience is treated throughout this review as a transversal breeding criterion rather than a constraint confined to a single section, and is revisited wherever a trait complex bears on stress interaction (Section 2.1, Section 4.3, Section 6 and Section 9). By integrating evidence from agronomy, plant physiology, soil ecology, rhizosphere microbiology and plant breeding, this review positions common bean improvement as a strategic lever for sustainable intensification and identifies priority breeding research directions for resilient organic cropping systems. We advance the working hypothesis that the agroecological functions of *P. vulgaris* beyond nitrogen fixation—namely rhizosphere microbiome recruitment, nutrient-use efficiency, soil carbon contribution and multi-stress resilience—are heritable, genotype-dependent trait complexes that can be deliberately selected for, rather than incidental by-products of agronomic management; this hypothesis is evaluated against the synthesized evidence and revisited explicitly in the Conclusions.

**Figure 1:**
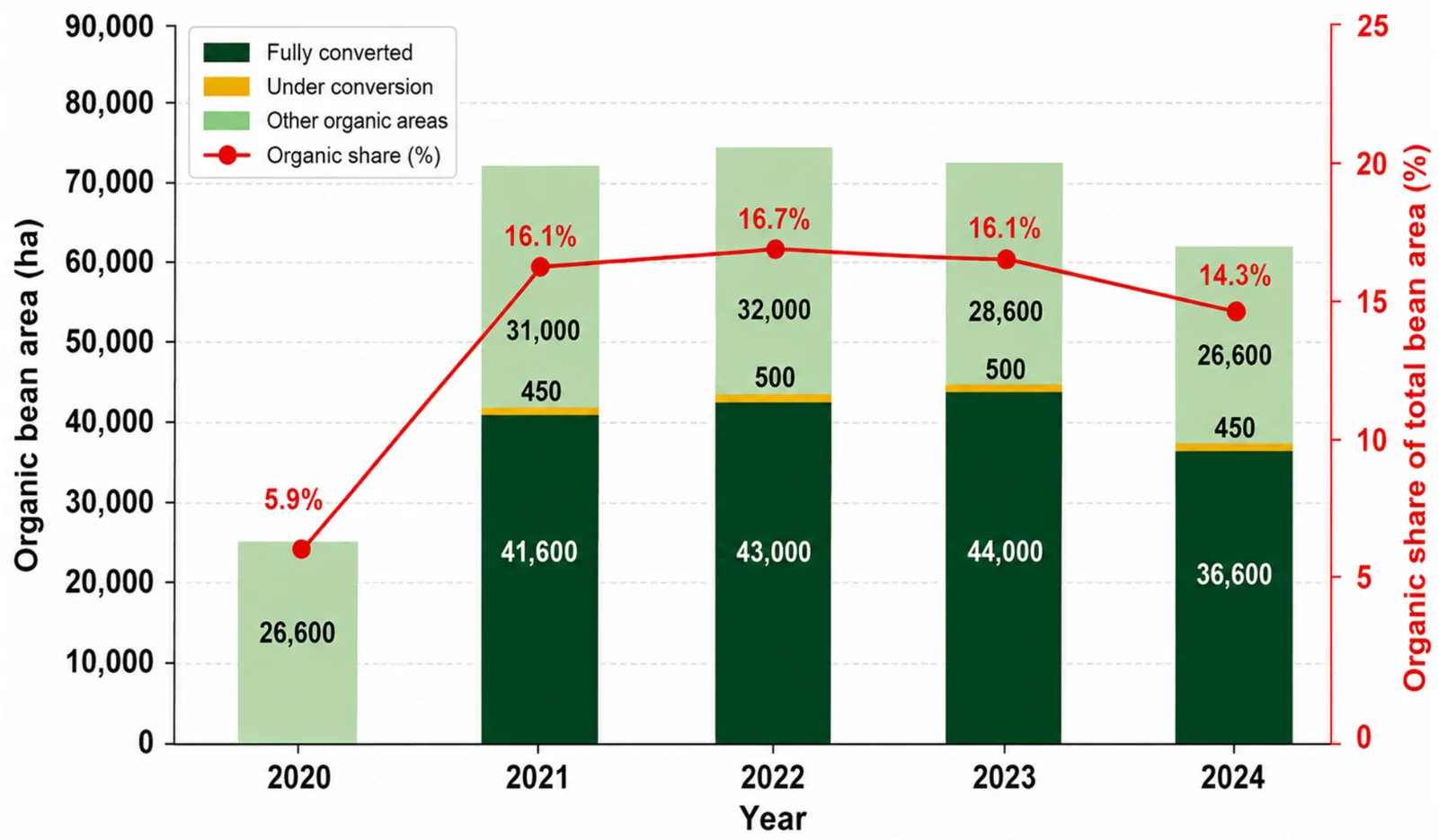
Trends in organic bean area under EU management (2020–2024). Stacked bars represent fully converted, under-conversion, and other organic area (ha); the red line denotes the share of total bean cultivated area under organic management. “Bean” refers to *Phaseolus vulgaris* grown for grain and fresh market use, as classified under Eurostat organic farming statistics; data source: Eurostat organic farming statistics.

Common bean is a cornerstone of global food security, particularly across Latin America and sub-Saharan Africa, supplying dietary protein, micronutrients and household income for hundreds of millions of people [5]. Cultivated across more than 20 million hectares, the species combines broad environmental adaptability with high nutritional value [6,7], while BNF reduces dependence on synthetic nitrogen and strengthens the resilience of low-input agriculture [8]. Advances in genomics, biofortification and rhizosphere ecology increasingly recognize common bean as a multifunctional genetic resource capable of supporting soil regeneration and adaptive capacity under climate change [9,10], repositioning it from a conventional food crop to a strategic target for breeding programs oriented toward ecosystem service delivery. Recent trends in the area under organic bean management are summarized in Figure 1.

**Figure 2:**
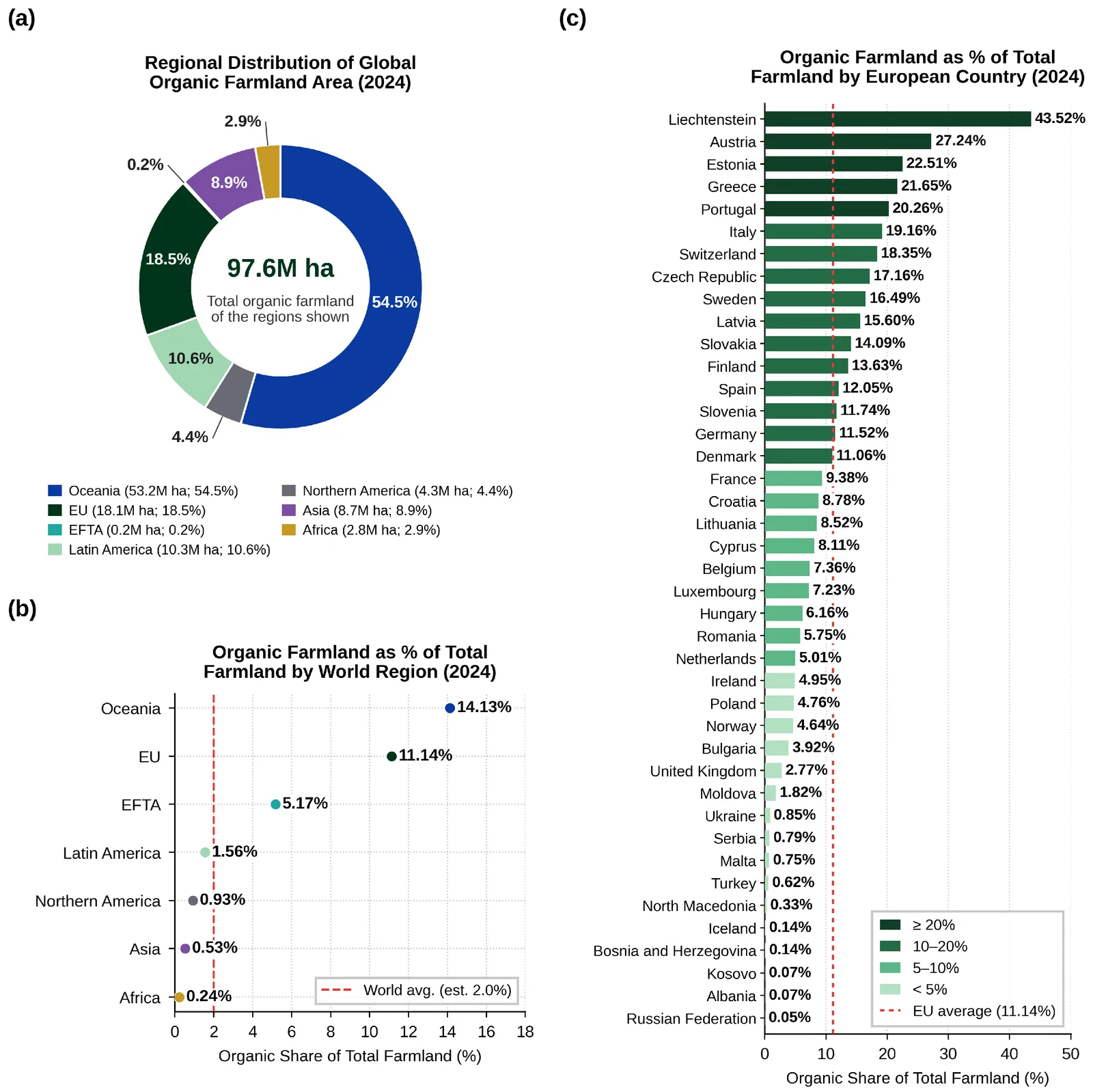
The global and European landscape of organic agriculture: regional distribution and country-level intensity of organic farmland adoption. (**a**) Regional distribution of organic farmland area (2024); the centre value is the total for the regions shown. (**b**) Organic farmland as a percentage of total farmland by world region (2024); the dashed line marks the estimated world average (2.0%). (**c**) Organic farmland as a percentage of total farmland by European country (2024), ranked in descending order and shaded by adoption class; the dotted line marks the EU average (11.14%). Data refer to total organic farmland across all crop types (not specific to *Phaseolus vulgaris*) and are compiled from the FiBL-IFOAM “The World of Organic Agriculture” report and Eurostat organic farming statistics.

Organic agriculture is a regulatory and scientific framework for sustainable production through the restoration of ecological processes rather than the intensification of external inputs [11], positioned within the European Union’s Green Deal, Farm-to-Fork and Biodiversity Strategies as a cornerstone of climate-neutral food systems [12,13], with certified organic farmland now exceeding 76 million hectares globally (Figure 2) [14,15]. Because organic systems substitute biological processes for chemical inputs, they constitute a distinct selective environment: persistent challenges including lower average yields, nutrient limitation and climate variability [16] define precisely the trait space, nutrient-use efficiency, weed competitiveness, and stress resilience that organic-adapted cultivars must occupy, yet remains largely unaddressed by conventional breeding pipelines.

For clarity, this review distinguishes management terms that are frequently used interchangeably in the literature but differ in scope and regulatory status. Organic agriculture denotes a certified production system governed by statutory standards (e.g., EU Regulation 2018/848) that prohibit synthetic inputs and most genetic-engineering techniques. Low-input agriculture is a broader, non-certified descriptor for systems that reduce, but do not eliminate, external chemical inputs. Regenerative agriculture emphasizes outcome-based soil and ecosystem restoration and currently lacks a harmonized legal definition. Agroecological management refers to the application of ecological principles to farming system design, encompassing but not limited to organic and regenerative practice. Climate-smart agriculture is an outcome-oriented framework prioritizing productivity, adaptation, and mitigation simultaneously, independent of input-use certification. Where this review draws on evidence generated under one of these frameworks to inform breeding priorities relevant to another, the source framework is specified explicitly. This distinction is particularly consequential for genome editing and related molecular breeding tools discussed in Section 4.4 and Section 7: Their compatibility with organic certification is not universal but varies by jurisdiction. For example, process-based exclusion under EU organic rules contrasts with product-based, case-by-case assessment in several non-EU regulatory systems, so that such tools should be regarded as organic-breeding-adjacent rather than uniformly organic-compliant.

The scientific relevance of *P. vulgaris* extends beyond biological nitrogen fixation: through dynamic interactions with rhizobia, arbuscular mycorrhizal fungi (AMFs) and plant-growth-promoting microorganisms, common bean regulates nutrient acquisition, carbon turnover and soil biological activity [4,17], positioning the species as a driver of ecosystem multifunctionality in low-input systems [18]. Because research has focused disproportionately on yield and nitrogen fixation, the mechanisms underpinning resilience and ecosystem service delivery remain insufficiently exploited for breeding. Emerging evidence indicates that common bean performance reflects coordinated interaction between plant genetics and its associated microbiome, i.e., a holobiont, offering new opportunities to enhance nutrient-use efficiency and stress tolerance through microbiome-informed breeding [19,20].

**Figure 3:**
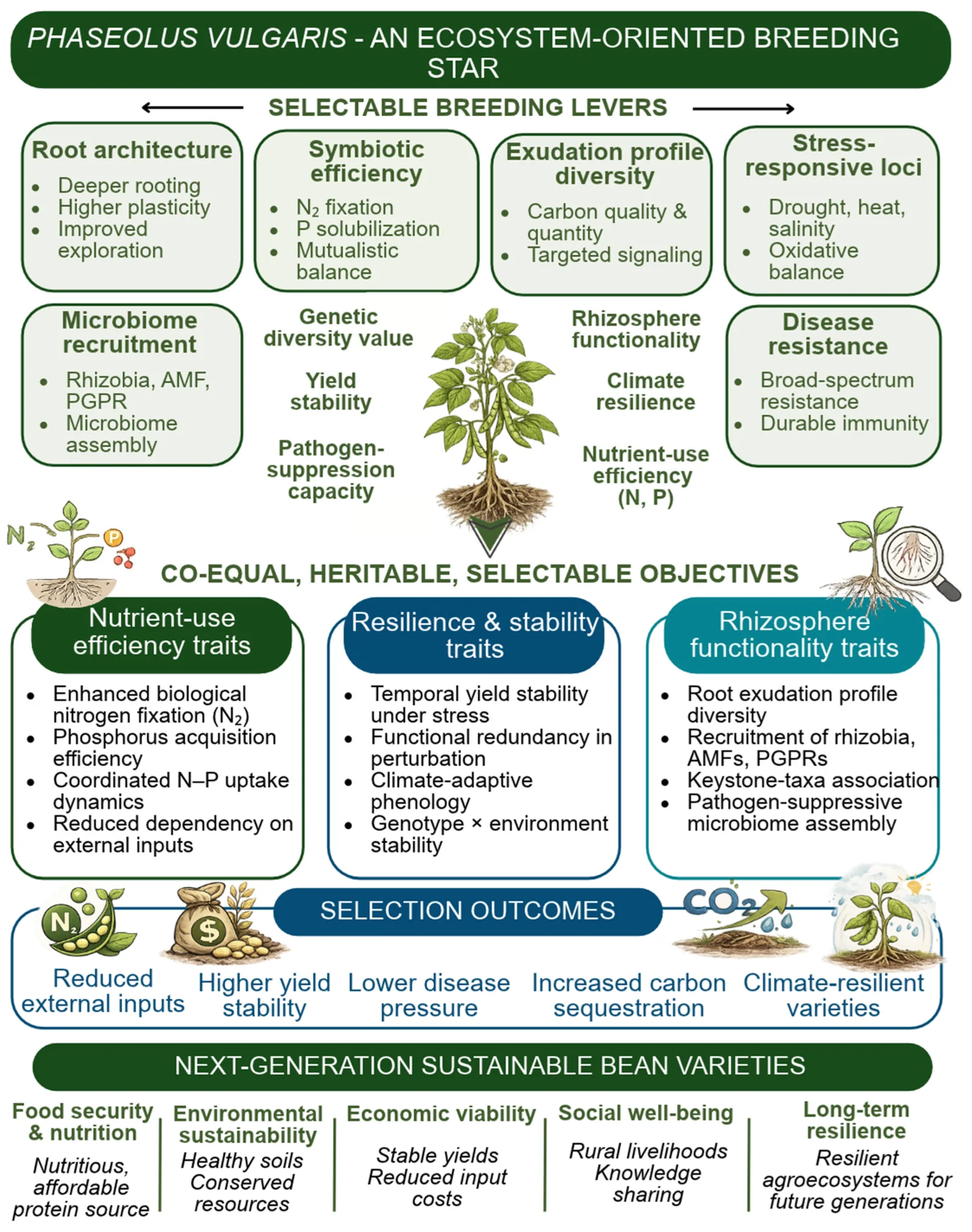
From trait-centric selection to ecosystem-oriented breeding in *Phaseolus vulgaris* L.: an integrated framework linking plant traits, rhizosphere functionality, resource-use efficiency, and agroecosystem resilience This is an original conceptual diagram created by the authors using Canva as a visual synthesis of the review text (not generated by an AI image tool). Abbreviations: N_2_, dinitrogen; P, phosphorus; AMFs, arbuscular mycorrhizal fungi; PGPRs, plant-growth-promoting rhizobacteria; N, P (nutrient-use efficiency), nitrogen and phosphorus.

This holobiont perspective provides the rationale for redefining breeding objectives beyond yield, disease resistance, and agronomic adaptation, to explicitly target nitrogen-fixation efficiency, root system architecture, rhizosphere recruitment capacity, nutrient-use efficiency, carbon sequestration potential and multi-stress resilience. Advances in quantitative genetics, high-throughput phenotyping, genomic prediction, pangenomics and microbiome-assisted breeding now enable identification of the polygenic traits governing not only productivity but also a genotype’s capacity to regulate belowground ecological processes. Integrating genomic information with rhizosphere ecology, systems biology, artificial intelligence and environmental phenotyping therefore represents a fundamental step toward next-generation, ecosystem-oriented cultivars, a paradigm shift from breeding for agronomic performance alone to breeding for ecosystem service delivery. Throughout this review, ecosystem-oriented breeding is used as the overarching term for this paradigm (Figure 3); holobiont-centred breeding (Section 8) designates its conceptual endpoint, in which host genotype and microbiome are selected as a coordinated unit; and microbiome-informed breeding denotes the more circumscribed set of methods, marker-assisted or genomic selection acting on microbiome-recruitment traits, through which that endpoint is operationally pursued.

## 2. Functional Biology, Nitrogen Fixation and Rhizosphere Engineering as Breeding Foundations

Because this synthesis draws on published evidence with varying phylogenetic proximity to the focal species, claims are qualified by evidentiary tier wherever such qualification is material to breeding inference: (i) direct experimental evidence obtained in common bean; (ii) evidence transferred by analogy from other legume species; and (iii) theoretical or general agroecosystem evidence not yet validated in this species. This tiering is made explicit wherever the risk of unwarranted cross-species or cross-scale generalization is greatest, particularly in the discussion of rhizosphere network function (Section 2.3) and of ecosystem-service co-determination (Section 3); elsewhere, the findings reported below derive from studies conducted directly in common bean unless another source is indicated.

### 2.1. Functional Biology as the Basis of Trait Selection

The ecological performance of *P. vulgaris* originates in its functional biology, which determines its capacity to acquire resources and interact with its biotic environment, providing the biological foundation for the symbiotic nitrogen fixation, rhizosphere assembly and ecosystem service delivery on which breeding must act [4,21]. Considerable phenotypic diversity within the species, determinate and indeterminate growth habits, contrasting canopy structures, and phenological variation, confer plasticity that allows genotypes to adjust biomass allocation and reproductive investment under fluctuating resource availability [22], an adaptive flexibility that is itself a selectable trait for resilience breeding. Belowground, root system architecture, rooting depth, lateral branching and root angle, directly regulate water uptake and phosphorus acquisition and are therefore primary determinants of resource-use efficiency wherever nutrient acquisition depends on biological rather than chemical processes [23,24]. Equally relevant to breeding is the species’ genetic competence for recruiting rhizobia, AMFs and beneficial rhizosphere microorganisms through coordinated root developmental programs and rhizodeposition [19], a competence governed by the same root architectural and exudation traits and therefore amenable to joint genetic selection. This functional integration is particularly consequential under combined stress, since genotypes capable of sustaining root growth and rhizosphere signalling under simultaneous drought and nutrient limitation consistently outperform those selected for single-stress tolerance alone [25,26], reinforcing the case for multi-stress phenotyping platforms as a breeding priority rather than sequential, single-factor stress screening [27].

### 2.2. Biological Nitrogen Fixation as a Selectable, Systems-Level Trait

BNF in *P. vulgaris* extends beyond its role as a nitrogen source, functioning as a systems-level process linking plant metabolism, microbial ecology and soil biogeochemistry [28,29]. Although common bean fixes less nitrogen than soybean or faba bean, its symbiosis with rhizobia remains a principal mechanism through which low-input agroecosystems reduce fertilizer dependence [30]. Nodulation success is governed by the interaction between host genotype, indigenous rhizobial diversity, and edaphic conditions, generating spatial variability in nitrogenase activity that is constrained less by the intrinsic capacity of elite inoculants than by their competitiveness within native microbial communities [31,32,33]. This host-genotype dependence identifies nodulation competitiveness, rather than fixation capacity alone, as an underexploited breeding target. Nodulation also restructures the rhizosphere through rhizodeposition and metabolite secretion that recruit beneficial microorganisms while suppressing pathogens [10,34], and nitrogen released through rhizodeposition and nodule decomposition sustains availability for subsequent crops [3,8,35]. Viewed from a breeding perspective, BNF is a multi-trait complex, comprising nodulation competitiveness, rhizodeposition capacity and post-senescence nitrogen release, rather than a single physiological function.

### 2.3. Rhizosphere Microbiome Assembly and Host Genetic Control

The rhizosphere of *P. vulgaris* is one of the most biologically active compartments of the agroecosystem, a plant-controlled interface in which root exudates selectively recruit microbial partners according to genotype and developmental stage. Critically for breeding, microbiome composition is strongly shaped by host genetics: domestication has altered root exudation patterns and the chemical signals governing microbial recruitment, meaning that breeding decisions already influence, often inadvertently, the rhizosphere microbiome that a cultivar assembles [10]. Within these assemblages, plant-growth-promoting rhizobacteria and AMFs perform complementary functions: phosphorus solubilization, siderophore production, induced systemic resistance and extraradical hyphal extension of the effective root absorptive surface, with co-inoculation generating synergistic effects that exceed single inoculants [36,37,38,39,40]. Organic systems support more connected, functionally redundant microbial networks than conventionally managed soils [41,42], and because network integrity, rather than the abundance of individual taxa, governs ecosystem functioning, breeding strategies should shift from selecting for single beneficial associations toward selecting host genotypes capable of sustaining resilient, functionally redundant microbiome assemblies. Recent metabolomic profiling of root exudate chemistry across bean genotypes confirms that exudate composition, rather than exudate quantity alone, determines which microbial functional guilds are recruited, a distinction with direct implications for marker development in microbiome-informed breeding [43].

A methodological caveat applies throughout this section: co-occurrence and correlation-based network analyses identify statistical association among microbial taxa, not necessarily causal regulation, direct interspecific interaction, or functional redundancy. Inferences regarding network connectivity and keystone taxa are therefore strongest when corroborated by functional-gene evidence, culture-based or gnotobiotic inoculation experiments, or validated synthetic-community (SynCom) assays; where only correlative network data are available, the corresponding conclusions are presented as hypothesis-generating rather than mechanistically established.

### 2.4. Soil Ecosystem Engineering: Root Traits Linking Aggregation and Carbon Sequestration

Root exudates and microbial metabolites facilitate the formation of stable macroaggregates that protect soil organic matter, coupling BNF with carbon sequestration and positioning root functional traits as regulators of soil ecosystem resilience [44]. Fine-root mechanical stabilization and mucilage-driven aggregate cohesion improve porosity and erosion resistance [45], while the comparatively low C:N ratio of bean residues favours the conversion of plant-derived carbon into mineral-associated organic matter—the most persistent soil carbon fraction—so that microbial processing rather than residue quantity alone governs long-term carbon stabilization [46,47,48]. This ecosystem engineering function extends to coordinated nitrogen–phosphorus cycling, since BNF together with the exudation of organic acids and phosphatases increase nutrient bioavailability [18,49], and adaptive root architecture further enhances phosphorus mobilization under limitation through rhizosphere acidification [50,51,52]. Root architectural and exudative traits governing aggregation and carbon persistence are consequently as breedable, and as agroecologically consequential, as canopy or seed traits.

### 2.5. Biodiversity Promotion as an Emergent, Genotype-Dependent Trait

*P. vulgaris* promotes biodiversity across organizational levels by creating ecological niches linking belowground microbial processes with aboveground trophic interactions [17,53]. Belowground, root exudation and plant–soil feedbacks regulate microbial recruitment and disease suppression [3,54], while aboveground, bean-based diversification enhances habitat heterogeneity for pollinators and natural enemies, so that biological control emerges as a system-level property of diversified food webs rather than of individual predator taxa [55,56,57]. These interactions are regulated by the genetic diversity of *P. vulgaris* itself: genotype-dependent variation in root architecture, phenology and rhizosphere chemistry links genetic diversity directly to ecosystem functioning [58], yet expression of these services remains constrained by landscape simplification and domestication-driven erosion of adaptive plant–microbe interactions [59]. This underscores that advancing sustainable intensification requires breeding strategies that explicitly prioritize ecological functionality alongside productivity [60,61].

The trait domains synthesized in Table 1 indicate, considered jointly, that biological nitrogen fixation, although historically the dominant lens for evaluating common bean’s agroecological value, accounts for only part of its multifunctional contribution. Macro- and micronutrient acquisition (phosphorus and potassium mobilization, Section 2.4), water-use efficiency (root-architecture-mediated hydraulic performance, Section 6), carbon allocation and sequestration (root-derived mineral-associated organic matter, Section 2.4), soil aggregation (mucilage-driven macroaggregate formation, Section 2.4), residual effects on subsequent crops (post-senescence nitrogen release and disease-suppressive legacy, Section 2.2 and Section 5.1), and nutritional and health-related functions (protein and micronutrient density, Section 3.1) are mechanistically distinct contributions that do not necessarily co-vary with nitrogen-fixation capacity; a genotype selected for superior biological nitrogen fixation is not thereby guaranteed to excel in water-use efficiency, carbon contribution or nutritional quality. Current breeding pipelines rarely evaluate these functions jointly, so that trade-offs and synergies among them remain empirically undercharacterized; treating them as a single multifunctionality index, rather than as covarying but partially independent trait domains, risks overstating the breeding progress achievable through nitrogen-fixation-centred selection alone.

## 3. Ecosystem Services as Emergent Breeding Targets

### 3.1. Provisioning and Supporting Services

Common bean delivers dietary protein, seed, feed, and income, particularly in smallholder systems [7,63], while biofortified cultivars reduce hidden hunger and support public health [63,64]. Beyond food provision, it generates income through grain and certified seed sales [65], and its residues provide biomass that strengthens circular, integrated production systems [66]. These provisioning services are underpinned by supporting services centred on soil fertility: symbiosis with *Rhizobium* supplies approximately 45–110 kg N ha^−1^ under field conditions [67], while rhizosphere interactions with AMFs and phosphate-solubilizing microorganisms mobilize phosphorus from insoluble mineral pools [8,49,50]. Because the magnitude of both provisioning and supporting services varies with genotype-specific nodulation, exudation, and residue quality, they should be treated as breeding-tractable outputs rather than fixed agronomic co-benefits.

Realizing this range of potential uses nonetheless entails trade-offs that a purely additive view of multifunctionality can obscure. Culinary, processing, and market-quality attributes are governed substantially independently of the agroecological traits emphasized elsewhere in this review, so that cultivars optimized for nitrogen-fixation efficiency, stress resilience, or rhizosphere recruitment are not automatically suited to a given end use, and the energy, water, and processing costs associated with realizing food-quality and health-related potential are seldom accounted for in assessments of the crop’s provisioning value. A critical appraisal of common bean’s potential uses therefore requires evaluating agroecological, nutritional and end-use quality traits as partially independent selection axes rather than assuming that gains in one domain translate automatically into gains across the others.

### 3.2. Regulating Services

The integration of common bean into rotations and intercrops improves carbon sequestration, moderates local climate, enhances biological pest and disease regulation, and strengthens water management, which are functions arising directly from BNF, root activity and microbial interactions [16,53,60]. Carbon sequestration increases as root biomass and rhizodeposits accumulate as soil organic carbon while BNF lowers the manufacturing emissions associated with synthetic nitrogen [67,68]. Biological pest suppression is strengthened by habitat complexity supporting predators and pollinators [55], while water regulation improves as root systems increase porosity and infiltration [59,69,70], and disease-suppressive soils arise from root exudates that stimulate rhizobia and AMFs antagonistic to soil-borne pathogens [54,71]. Field microclimate regulation, reduced soil surface temperature, and moderated evaporation under bean canopies further contribute to local climate buffering during dry periods [72,73]. The regulatory functions governing nutrient cycling, biological interactions, and ecosystem resilience are mechanistically rooted in root-architectural, symbiotic, and rhizosphere-associated traits, rendering them measurable and, in part, heritable and selectable phenotypic expressions of ecosystem-oriented cultivars rather than purely incidental ecological externalities; because carbon sequestration, pest regulation, water regulation and microclimate buffering are jointly co-determined by crop-rotation design, intercropping configuration, landscape structure and soil management, the genotype-attributable component of each service should be regarded as established only where genotype-controlled trials, rather than observational comparisons, provide supporting data.

### 3.3. Cultural, Socio-Economic and Circular Bioeconomy Roles

Common bean supports agricultural heritage through landraces maintained by smallholder and indigenous farmers, particularly women, who preserve genetic diversity adapted to local conditions [74,75], while seed-exchange networks and participatory variety selection conserve germplasm and transfer indigenous knowledge across generations [76], and income generated through certified seed sales and local and emerging organic value chains strengthens rural livelihoods [77,78,79]. Residues additionally advance the circular bioeconomy: pods, stems and leaves provide nitrogen-rich biomass for composting and green manure that reduces synthetic fertilizer dependence while improving organic matter and structural stability [66,80], and residue incorporation enhances carbon sequestration while suppressing weeds and soil-borne disease [81,82,83,84]. Research gaps remain regarding residue-mineralization dynamics and standardized composting protocols across agroecological conditions [85,86,87], and closing them will require integrating microbial ecology with breeding for residue quality traits. These service categories and their genetic basis are summarized in Table 2.

## 4. Constraints as Breeding Priorities

### 4.1. Weed Competitiveness

Weed competition remains a principal constraint on common bean performance, particularly during the first 30–45 days after emergence when slow early growth and delayed canopy closure reduce competitive ability [89]. Current research advocates agroecological weed management, diversified rotations, living mulches and cover crops that suppress weeds through physical and allelopathic mechanisms [90,91], complemented by mechanical strategies that preserve soil structure and beneficial microbial communities [92,93]. The effectiveness of these interventions depends strongly on species selection, planting density, and establishment timing relative to bean phenology [94,95], and weed pressure during the critical early period can produce irreversible yield losses if canopy closure is delayed beyond the crop’s competitive threshold [96,97]. Mechanical strategies, including shallow cultivation and strategic tillage timing, further disrupt weed emergence while preserving beneficial microbial communities [98]. From a breeding perspective, however, these management interventions substitute for a trait that is rarely selected directly—early vigour and canopy closure rate—which should be treated as an explicit ideotype target for organic-adapted cultivars rather than solely a management problem.

### 4.2. Biotic Constraints and the Case for Durable, Polygenic Resistance

Productivity is constrained by biotic stresses including anthracnose (*Colletotrichum lindemuthianum*), common bacterial blight (*Xanthomonas axonopodis* pv. *phaseoli*), white mold and soil-borne *Fusarium* and *Pythium* species [99,100]. Historical reliance on resistant cultivars and synthetic pesticides has been eroded by rapid pathogen evolution [101], shifting emphasis toward integrated biological management combining host resistance with microbial inoculants and improved agronomic practice [10,102]. Widespread implementation nonetheless remains constrained by inconsistent field performance and an incomplete understanding of plant–microbiome interactions [103], so that future breeding should integrate genomics and microbiome engineering to strengthen durable, polygenic resistance rather than relying on single-gene resistance sources prone to being overcome. Recent multi-omics characterization of pathogen diversity and host–microbiome interactions is beginning to clarify which resistance mechanisms are transferable across production systems [104,105], while formulation and delivery constraints continue to limit consistent field performance of biological control agents [106,107].

Arthropod pests, including bean weevils, aphids, thrips and spider mites, further reduce yield and grain quality, and current research favours Integrated Pest Management emphasizing conservation biological control and host-plant resistance over chemical dependence [101,108]. Intercropping and diversified landscapes enhance predator and parasitoid populations [109], while resistant varieties, hermetic storage and push–pull systems complement these approaches [110,111]; future progress requires integrating ecological monitoring with molecular tools to develop locally adapted, breeding-informed IPM strategies [112], engaging farmer participatory networks and landscape-scale coordination to sustain natural enemy communities across cropping seasons [113,114,115].

### 4.3. Nutritional and Climate-Induced Abiotic Constraints

Performance is fundamentally constrained by phosphorus, potassium and micronutrient deficiencies that limit growth, BNF and yield stability [116], with phosphorus availability frequently restricted by fixation with iron, aluminum and calcium in weathered soils, and potassium deficiency reducing water-use efficiency and nodule function [117]. Agroecological nutrient management combines organic amendments with rhizobia, AMFs, and phosphate-solubilizing bacteria to enhance mobilization [118,119,120], yet the underlying nutrient-acquisition efficiency remains genotype-dependent and insufficiently exploited in breeding pipelines, particularly given that potassium and micronutrient deficiencies compound phosphorus limitation under the highly weathered, low-fertility soil conditions typical of many organic smallholder systems [121,122,123].

Climate change compounds these limitations: drought impairs stomatal conductance, root development and BNF [25]; heat stress causes pollen sterility and flower abortion [124]; and flooding induces root hypoxia and nodule suppression [125], with combined stresses imposing greater physiological damage than any single factor [126,127]. Climate-resilient improvement increasingly integrates high-throughput phenotyping, QTL mapping and multi-omics to identify stress-responsive traits, deeper rooting, osmotic adjustment, canopy temperature regulation [22,128,129], complemented by introgression of adaptive alleles from wild relatives such as *Phaseolus acutifolius*, CRISPR/Cas9 editing and AI-assisted genomic prediction [130,131,132,133].

### 4.4. The Organic Seed and Breeding Bottleneck

The agroecological potential of common bean in organic farming is constrained by the limited availability of cultivars bred for low-input environments and a persistent shortage of certified organic seed [134]. Most commercial varieties were developed for conventional, high-input agriculture and lack traits essential for organic production, nutrient acquisition efficiency, weed competitiveness and stress resilience [10,135,136], while breeding programs have largely overlooked traditional landraces despite their superior diversity and local adaptation [137]. Certification frameworks emphasizing distinctness, uniformity and stability further restrict the commercialization of genetically diverse populations. Participatory plant breeding, decentralized seed production and community seed banks have consequently emerged as critical strategies for integrating farmer knowledge with formal breeding to develop locally adapted cultivars while conserving genetic diversity [138], an approach that supportive policy and greater investment in legume breeding must reinforce. These constraints and their corresponding breeding targets are summarized in Table 3.

## 5. Agroecological Management as the Selective Environment for Ecosystem-Oriented Cultivars

### 5.1. Crop Rotation and Intercropping

Crop rotation improves nitrogen economy and disease suppression by integrating common bean into diversified sequences that promote BNF and reduce synthetic fertilizer dependence [35,61,139,140], reinforced by legume-residue decomposition that stimulates microbial activity and phosphorus mobilization [45,141,142]. Rotations create disease-suppressive soils by disrupting host-specific pathogen cycles such as *Fusarium oxysporum* while promoting antagonistic *Bacillus* and *Pseudomonas* taxa [143,144,145,146]. Effectiveness nonetheless remains context-dependent, since microbial responses vary with residue quality, soil type and management intensity [147,148], and important gaps remain regarding synchronization between nitrogen release and crop demand under changing climate [149,150].

Intercropping consistently demonstrates superior biological productivity relative to monoculture, with Land Equivalent Ratio (LER) values frequently exceeding 1.2 in bean–cereal systems [1,151], reflecting the efficient partitioning of light, water and nutrients rather than simple additive effects. Complementary indices, Competitive Ratio, Aggressivity and Actual Yield Loss reveal that cereals often dominate early growth while common bean compensates through enhanced BNF, so that competition transitions toward facilitation at later developmental stages [152,153,154,155,156]. These dynamics are driven by resource complementarity: common bean develops shallow, rhizosphere-acidifying roots that increase phosphorus availability for neighbouring crops, while companion cereals exploit deeper soil layers [51,157,158], and contrasting canopy architectures improve radiation-use efficiency and water productivity relative to monocultures [159,160,161]. Because both LER and its component indices are strongly genotype-dependent, breeding for complementary root and canopy architecture, rather than for sole-crop performance alone, would substantially extend the ecological value of intercropping systems. Realizing these benefits at scale nonetheless depends on site-specific optimization: rotation and intercrop designs that perform well under one pedoclimatic regime frequently underperform elsewhere, requiring long-term, multi-location trials that couple agronomic performance with socioeconomic feasibility assessments before recommendations can be generalized across regions [162,163,164,165].

### 5.2. Cover Cropping, Organic Fertilization, Biostimulants and Biopesticides

Legume, grass and living-mulch cover crops complement, rather than substitute for, one another: legumes contribute 50–320 kg N ha^−1^ through symbiotic fixation [166], while grasses generate high-biomass residues that improve aggregate stability, and grass–legume mixtures outperform single-species covers by combining rapid nitrogen release with prolonged residue persistence [167,168]. Adoption nonetheless remains constrained by labour requirements and limited seed access [169,170]. Species selection and termination timing further determine whether cover crops synchronize nitrogen release with bean demand or instead compete for water and light during establishment [171,172], while broader adoption is additionally shaped by land tenure arrangements and short-term profitability incentives that discourage multi-year investment in soil-building rotations [173].

Organic fertilization strategies, vermicompost, farmyard manure, and biochar significantly improve productivity by enhancing nutrient cycling and microbial activity [174,175]. Biochar increases cation exchange capacity and nutrient retention while creating stable microsites that improve *Rhizobium*–legume symbiosis [176,177,178,179], and co-application with compost consistently outperforms individual amendments [180]. Integrated nutrient management combining organic amendments with reduced mineral inputs frequently matches conventional fertilization while improving nodulation and drought resilience [120,181,182], effects driven by amendment-stimulated bacterial and mycorrhizal activity [45,183]. Seaweed extracts, humic substances, protein hydrolysates, and microbial or botanical biopesticides can further enhance agroecological crop performance by promoting stress acclimation, stimulating physiological resilience, and strengthening endogenous mechanisms of pathogen suppression. Notably, several of their modes of action converge with the biological processes governing root–rhizosphere interactions and the recruitment of beneficial microbial consortia, suggesting that genotypes with intrinsically high rhizosphere-recruitment capacity may exhibit reduced dependence on externally supplied biostimulant and biopesticide inputs. This convergence highlights the potential for integrating plant genetic capacity for microbiome recruitment with targeted biological inputs as a complementary breeding and crop-management strategy for reducing external input requirements while maintaining resilience and productivity.

### 5.3. Biostimulants and Biopesticides as Complementary Inputs to Intrinsic Rhizosphere-Recruitment Capacity

Biostimulants, including seaweed extracts, humic substances, protein hydrolysates and amino-acid formulations, enhance nutrient-use efficiency, stress tolerance and root development in common bean through hormone-like and antioxidant mechanisms [184,185], with recent field evaluations confirming consistent gains in nodulation and stress recovery under organic management [186]. Microbial and botanical biopesticides, entomopathogenic fungi, *Bacillus*- and *Trichoderma*-based formulations, and plant-derived extracts complement host resistance by suppressing pathogens through antibiosis, competition and induced systemic resistance [104,105,106], with efficacy strongly influenced by formulation stability and strain persistence in the field [187,188]. Because both technologies primarily function by supplementing or amplifying key rhizosphere-mediated processes—including rhizobial competitiveness, induced systemic resistance, and root vigour—genotypes characterized by intrinsically strong recruitment of beneficial microbial consortia may achieve comparable levels of stress protection and pathogen suppression with substantially lower external biostimulant and biopesticide inputs. This functional complementarity therefore positions such products not as substitutes for the inherent biological capacity of the plant–microbiome system, but rather as bridging and complementary interventions that can enhance the performance of ecosystem-oriented cultivars during the transition toward more self-regulating, low-input production systems (practices summarized in Table 4) [189,190].

## 6. Resource-Use Efficiency as a Composite Breeding Objective

Nitrogen-use efficiency (NUE) in organic common bean depends primarily on BNF, soil organic matter mineralization and nutrient recycling rather than synthetic inputs [2], yet BNF effectiveness is strongly influenced by soil moisture, phosphorus availability and genotype-strain compatibility, producing considerable variability in nitrogen acquisition [116,191]. Locally adapted rhizobial inoculants and stress-tolerant cultivars substantially enhance fixation [192,193], while cereal–legume intercropping and rotation improve nitrogen cycling and reduce losses [152]. Genotype-specific differences in nodule senescence timing and nitrogen remobilization efficiency further explain much of the variability observed across field trials, indicating that NUE improvement should target the full nodulation-to-remobilization sequence rather than fixation capacity alone [182,183]. Maximizing NUE requires balancing the carbon cost of symbiotic fixation against biomass production, prioritizing cultivar-specific nitrogen-use traits and root architecture as targets for phenotyping and isotopic characterization [194,195].

Water-use efficiency (WUE) is similarly a determinant of productivity under climate variability, improved primarily through soil organic matter, aggregate stability and hydraulic conductivity that increase infiltration while reducing evaporation [196,197]. Deficit irrigation success depends on precise scheduling, since excessive restriction during flowering can reduce nodulation and yield [198], while AMFs and drought-tolerant rhizobia contribute to improved hydraulic conductivity, though field performance requires further validation [199,200]. Practical implementation should combine precision irrigation with locally adapted drought-tolerant cultivars [201,202], and future WUE metrics should integrate soil biological properties and microbial function [203]. Reduced-tillage and mulching practices further conserve moisture and moderate rhizosphere temperature, extending the window during which nodulation and nitrogen fixation remain physiologically active under progressive drought [204].

Resource-use efficiency and carbon footprint assessments demonstrate that, although organic systems often yield less than conventional ones, reliance on BNF and diversified rotations substantially lowers fossil energy consumption and greenhouse gas emissions [205,206], with legume integration into conservation-oriented rotations improving soil organic carbon storage [1,207]. Trade-offs remain between maximizing yield and preserving soil carbon, since intensive residue removal can offset sequestration gains unless complemented by conservation tillage and precision nutrient management [152,208], while environmental performance depends on site-specific conditions that require localized emission factors and dynamic life-cycle assessment models [209,210,211,212]. Life-cycle comparisons across contrasting soil types and rotation designs consistently show that legume-based systems reduce net greenhouse gas intensity relative to synthetic-input-reliant systems, though quantification remains sensitive to system boundary assumptions and regional emission factors [213].

Energy efficiency benefits from BNF’s substantial reduction in dependence on the energy-intensive Haber–Bosch process [30], further enhanced by livestock manure integration and rhizobial inoculation [194], though partially offset by increased mechanical weed control [214,215]. Site-specific phosphorus application and reduced mechanical passes further improve the overall energy balance of organic rotations without compromising nodulation performance [216,217]. Circular nutrient management through recycled residues, composts and manures reduces synthetic fertilizer dependence [218], yet maintaining balanced phosphorus and potassium availability remains challenging when grain-harvest nutrient removal exceeds replenishment [219], requiring standardized quality assurance and improved synchronization between mineralization and crop demand [220,221,222,223], alongside logistical and economic trade-offs in transport and processing that vary considerably by region and farm scale [224]. Comparative assessments across cropping systems further indicate that legume-integrated rotations achieve superior nutrient-use efficiency and reduced input dependency relative to cereal monocultures, particularly where residue and manure recycling are locally coordinated. Standardized bio-fertilizer quality protocols and improved kinetic modelling of nitrogen release from recycled residues remain priority research needs for scaling circular nutrient management beyond pilot demonstrations [225].

Nitrogen- and water-use efficiency are physiological metrics expressed at the plant or canopy scale and share a common biological basis in root system architecture, symbiotic efficiency and rhizosphere recruitment, rendering them directly amenable to genotype-level selection. Carbon footprint, energy efficiency and life-cycle environmental performance, by contrast, are emergent properties of the farm or cropping-system scale, co-determined by management practice, input logistics, and system-boundary assumptions, and are therefore breeding-relevant chiefly insofar as the underlying plant traits reduce system-level input dependency, rather than being themselves directly selectable traits. Resource-use efficiency is consequently best treated as a multi-level construct, comprising a genotype-selectable physiological tier (nitrogen- and water-use efficiency) and a management-mediated system-performance tier (carbon and energy footprint), rather than a single, undifferentiated trait complex.

## 7. Emerging Technologies for Ecosystem-Oriented Breeding

Precision agriculture integrates IoT-enabled sensing, geospatial analytics and machine learning to enable the site-specific optimization of nutrient, water and pest management, enhancing nitrogen-use efficiency while strengthening soil ecological processes [226], with future progress depending on interoperable, cost-effective, AI-enabled frameworks accessible to organic smallholders [227]. Remote sensing, integrating multispectral, hyperspectral and UAV-based platforms with artificial intelligence, enables the non-destructive quantification of canopy architecture, water status, and nutrient-use efficiency, supporting high-resolution prediction of genotype × environment responses [228,229]. Artificial intelligence further couples phenomic, environmental, and agronomic datasets for early disease diagnosis and yield forecasting while reinforcing BNF and climate-resilience assessment [230,231], with recent multimodal sensor-fusion frameworks demonstrating improved transferability across heterogeneous organic field conditions [232].

High-throughput and digital phenotyping redefine breeding targets beyond yield to ecosystem functioning: UAV- and ground-based phenomics, hyperspectral imaging, and enviromics enable the non-destructive characterization of root architecture, nutrient-use efficiency, BNF, and stress adaptation [233], and coupling phenomics with genomics substantially enhances predictive breeding and ideotype design while reducing phenotyping bottlenecks [234]. Molecular breeding—integrating marker-assisted selection, genome-wide association studies, genomic selection and pangenomics with high-throughput phenotyping and envirotyping—enables the identification of loci controlling nutrient-use efficiency, root architecture and plant–microbiome interactions under low-input organic systems [233,235], while future breeding paradigms should explicitly model genotype × environment × microbiome interactions and epigenetic regulation to accelerate resilient cultivar development while preserving organic certification integrity [234].

Genome editing enables precise modification of loci controlling drought tolerance, nutrient-use efficiency and nitrogen fixation without the linkage drag of conventional breeding [236], though implementation is constrained less by technical limitation than by divergent regulatory frameworks contrasting process-based organic certification with product-based assessment of transgene-free edited cultivars [237,238]; future governance should integrate molecular traceability, transparent risk assessment and equitable seed sovereignty to reconcile innovation with agroecological principles [239,240]. Microbiome engineering is replacing single-strain inoculants with functionally assembled synthetic microbial communities (SynComs) that exploit metabolic complementarity to enhance nitrogen fixation, phosphorus mobilization and disease suppressiveness under heterogeneous field conditions [241,242], with metagenomics and computational community design enabling the predictive optimization of plant–microbiome interactions [243]; future implementation requires standardized, organic-compliant SynComs that preserve native microbiome functionality [244], with early field trials of designed consortia reporting improved colonization persistence relative to single-strain inoculants under variable soil moisture and temperature.

Climate-smart breeding integrates genomics, phenomics and multi-environment testing to accelerate identification of genotypes resilient to interacting heat and drought stresses while preserving productivity under low-input organic management. Exploiting landraces, wild relatives, and *Phaseolus acutifolius* germplasm expands adaptive allelic diversity controlling root architecture, water-use efficiency and nitrogen fixation [245], while multi-omics, GWAS and genomic selection enable dissection of complex genotype × environment × management interactions [246], and future breeding should integrate physiological plasticity with beneficial rhizosphere interactions to stabilize ecosystem functions under increasingly variable climatic conditions [18]. This direction is reinforced by recent envirotyping studies linking genotype-specific canopy and root responses to multi-year climate records across contrasting production regions. Complementary trait-mapping efforts targeting reproductive-stage heat tolerance and combined stress recovery further indicate that resilience under organic, low-input management is governed by largely independent genetic loci from those controlling yield under optimal conditions, reinforcing the case for dedicated organic-adapted selection programs rather than the direct transfer of conventionally bred cultivars.

Collectively, these technologies constitute tiers of an integrated breeding pipeline rather than independent tools: precision agriculture and remote sensing generate the high-resolution environmental and phenotypic data streams that calibrate high-throughput and digital phenotyping; phenomic outputs are in turn coupled with genomic, transcriptomic and metabolomic data through molecular breeding and genomic selection to resolve the genetic architecture of resource-use efficiency and rhizosphere-recruitment traits; genome editing and microbiome engineering then translate the loci and microbial functions identified at that stage into deployable cultivars and compatible SynComs; and climate-smart, multi-environment testing validates the resulting genotype–microbiome combinations under the combined-stress conditions representative of organic field environments. Artificial intelligence functions as the connective layer across this pipeline, integrating phenomic, genomic, environmental and microbiome data streams to prioritize candidate traits and accelerate selection decisions at each stage, rather than operating as a discrete, freestanding technology.

## 8. Conceptual Synthesis: From a Yield-Centred to a Holobiont-Centred Breeding Paradigm

Organic management reshapes the common bean rhizosphere through interconnected processes that regulate microbial assembly and ecosystem functioning: crop rotation, intercropping and residue retention modify root exudation patterns that selectively recruit functionally specialized microbial consortia [42], governing communities enriched in rhizobia, AMFs and PGPRs that collectively increase nitrogen fixation and pathogen suppression [247]. These functions emerge from ecological interaction rather than taxonomic composition alone: keystone taxa and highly connected networks provide functional redundancy [42,148], while root-derived metabolites regulate microbial signalling in dynamic feedback loops that influence nodulation efficiency and defence pathways [183,248]. Future management should transition from manipulating microbial abundance toward engineering functional rhizosphere networks through systems biology [54,249], positioning the rhizosphere as a programmable ecological interface, one in which host genotype, not management alone, is the ultimate lever of control. As in Section 2.3, this interpretation of network connectivity and keystone taxa rests predominantly on correlative co-occurrence data and should be read as hypothesis-generating pending functional and SynCom-based validation.

Organic bean agroecosystems operate through dynamic trade-offs rather than the maximization of individual agronomic components. BNF reduces external nitrogen requirements yet increases dependence on phosphorus, making P availability the principal regulator of nodule function [3,250], so sustainable productivity depends on coupling nitrogen fixation with enhanced phosphorus mobilization through diversified rotations and mycorrhizal association [251]. Increasing plant diversity strengthens resource complementarity and pest regulation, yet may intensify early-season weed competition if crop architecture and temporal niche occupation are poorly synchronized [1,90], while replacing synthetic inputs with ecological processes transfers system regulation to biological interactions, increasing management complexity but strengthening resource-use efficiency and long-term resilience [252]. Although organically managed beans generally achieve lower maximum yields than conventional systems, diversified agroecosystems consistently exhibit greater temporal yield stability because functional redundancy and complementary resource capture buffer environmental perturbation [11,253,254].

**Figure 4:**
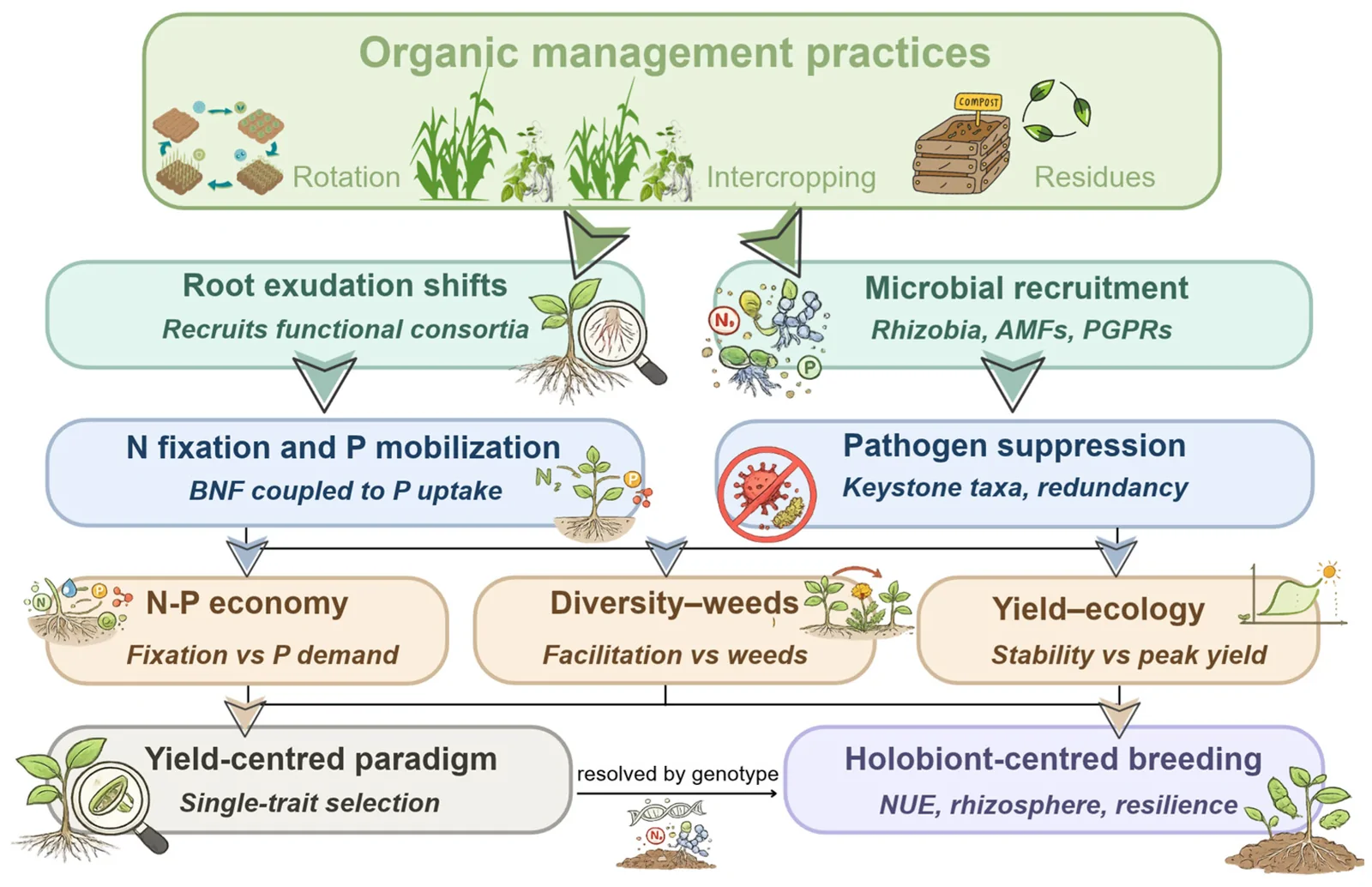
From organic management to holobiont-centred breeding: an integrative framework linking management practices, rhizosphere processes, ecosystem functions, and genotype-dependent agroecological performance of *Phaseolus vulgaris* L. This is an original conceptual diagram created by the authors using Canva as a visual synthesis of the review text (not generated by an AI image tool). Abbreviations: N, nitrogen; P, phosphorus; N_2_, dinitrogen; BNF, biological nitrogen fixation; AMFs, arbuscular mycorrhizal fungi; PGPRs, plant-growth-promoting rhizobacteria; NUE, nutrient-use efficiency.

The conceptual synthesis advanced in this review, linking management practices to rhizosphere processes and ecosystem service delivery, reveals that the trade-offs long assumed to constrain organic bean production, between nitrogen and phosphorus economy, between plant diversity and weed competition, and between yield and ecological functionality, are not fixed biological constraints, but management- and genotype-dependent outcomes that coordinated breeding and agroecological design can substantially resolve [157,255]. Common bean’s capacity to function as a regulator of regenerative, climate-resilient agroecosystems is therefore neither incidental nor immutable, but a trait complex amenable to deliberate genetic improvement [52,256,257], one in which resource partitioning and biological redundancy stabilize productivity while reducing nutrient losses and external energy requirements [53,61,109,258]. Recent multi-site evidence spanning contrasting rotation designs and cover-crop mixtures reinforces this conclusion, showing that complementarity effects, rather than any single input substitution, drive the resilience gains attributed to organic bean systems [259,260,261]. Comparable convergence is reported for biofertilizer and microbial-consortium trials evaluated across independent research groups and production regions [262,263,264], while recent syntheses of long-term organic trial networks corroborate that these complementarity effects persist, and in several cases strengthen, over successive cropping cycles [265,266]. Ultimately, the knowledge gaps identified across agronomy, soil ecology, plant physiology and rhizosphere microbiology converge on a single strategic imperative for the breeding community: common bean improvement must transition from a yield-centred paradigm to one that treats nutrient-use efficiency, rhizosphere functionality and climate resilience as co-equal, heritable, selectable objectives. This holobiont-centred synthesis is summarized in Figure 4.

## 9. Knowledge Gaps and Priority Breeding Research Directions

Priority research should optimize diversified rotations and intercropping to synchronize nutrient mineralization with crop demand while enhancing weed suppression [3,86], supported by process-based nitrogen models quantifying rotation legacy effects across pedoclimatic environments [8]. In soil ecology, research should mechanistically quantify how root-derived carbon inputs regulate microbial diversity and long-term carbon stabilization [256], integrating soil physics with metagenomics and isotope tracing to identify biological drivers of carbon persistence and disease suppressiveness beyond residue decomposition alone.

In plant physiology, future studies should elucidate mechanisms controlling phosphorus acquisition, nitrogen-fixation efficiency and carbon allocation under low-input environments through integrated phenomics [267,268], emphasizing root architectural plasticity to identify ideotypes combining efficient nutrient acquisition with drought and heat tolerance [25,135]. Rhizosphere microbiology should transition from descriptive microbiome characterization toward predictive understanding of plant–microbe interactions governing nutrient cycling and stress resilience [248], providing the mechanistic insight required to engineer synthetic microbial consortia and microbiome-informed breeding strategies. For climate adaptation, research should integrate genomics, transcriptomics and climate modelling to identify adaptive mechanisms conferring tolerance to combined heat and drought stresses rather than isolated stress factors [269,270], evaluating microbial inoculants as complementary tools under projected climate scenarios [271,272].

Water management research should optimize biologically driven strategies integrating precision irrigation, cover crops and AMFs to improve hydraulic conductivity and drought resilience [273], while digital agriculture should develop affordable decision-support platforms integrating UAVs and IoT networks for real-time monitoring, with algorithms specifically calibrated for organic cropping systems [274,275,276,277]. Above all, plant breeding priorities should target low-input adaptation by integrating genomic selection, high-throughput phenotyping and participatory breeding to identify genotypes combining superior nutrient-use efficiency, root plasticity, BNF and competitive ability within diversified cropping systems [135]; explicitly incorporating plant–soil feedbacks, microbiome recruitment, and intercropping compatibility; and exploiting landraces and wild relatives to restore adaptive traits diminished during conventional high-input breeding [25]. Finally, socio-economic and policy research should strengthen regional value chains, decentralized seed systems and payments for ecosystem services that improve the competitiveness of organic legumes [53,278], through multi-actor innovation platforms linking farmers, breeders and policymakers to evaluate business models and True Cost Accounting approaches capable of internalizing environmental benefits (priority directions summarized in Table 5) [279].

## 10. Conclusions

The working hypothesis advanced in the Introduction, namely that the ecosystem functions of common bean beyond nitrogen fixation constitute heritable, genotype-dependent trait complexes amenable to deliberate selection rather than incidental by-products of management, is confirmed by the evidence synthesized across Section 2, Section 3, Section 4, Section 5, Section 6, Section 7, Section 8 and Section 9: common bean’s agroecological value extends far beyond nitrogen fixation, encompassing rhizosphere microbiome engineering, soil carbon stabilization, biodiversity support and multiple regulating ecosystem services, all of which are mechanistically rooted in root architectural and exudative traits that are demonstrably heritable. The evidence synthesized here indicates that the trade-offs long assumed to limit organic bean production, between nitrogen and phosphorus economy, between plant diversity and weed competition, and between yield and ecological functionality, are not fixed biological constraints but management- and genotype-dependent outcomes. Realizing common bean’s potential as a regenerative, climate-resilient crop therefore requires reconceptualizing the species as a holobiont and explicitly selecting for nitrogen-fixation efficiency, rhizosphere recruitment capacity, nutrient-use efficiency and multi-stress resilience alongside conventional agronomic traits.

This reframing carries practical implications for three interdependent communities. For breeders, it argues for incorporating rhizosphere- and microbiome-related phenotypes into routine selection pipelines alongside yield and disease resistance. For agronomists and organic producers, it clarifies that management practices such as rotation, intercropping and biofertilization are most effective when matched to genotypes bred for complementary root and rhizosphere traits, rather than treated as universally transferable recommendations. For policymakers, it underscores that seed-system and certification frameworks calibrated for uniform, high-input cultivars constrain the availability of the very genetic diversity that organic and regenerative systems require. Achieving this transition will require sustained investment in phenotyping infrastructure, genomic resources and interdisciplinary collaboration across breeding, microbiology and agroecology, positioning common bean breeding at the forefront of the transition toward regenerative, climate-smart organic agriculture.

## Figures and Tables

**Table 1:** Functional trait domains of *Phaseolus vulgaris* L. relevant to ecosystem-oriented breeding.

Trait Domain	Underlying Mechanism	Breeding Relevance	Key References
Root system architecture	Basal root angle, lateral branching, root hair density regulating topsoil P foraging vs. subsoil water access	Trade-off between drought and P-deficiency tolerance is genotype-specific and selectable	[23,24]
Nodulation competitiveness	Flavonoid–NodD signalling; host-strain compatibility under indigenous rhizobial competition	Nodulation success depends on genotype × strain compatibility, not inoculant quality alone	[31,32,33]
Rhizosphere recruitment capacity	Root exudate composition (organic acids, flavonoids, mucilage) shaping PGPR and AMF assembly	Host genetics already shapes microbiome composition; a latent, unselected breeding target	[10]
Phosphorus mobilization	Rhizosphere acidification, phosphatase secretion, carboxylate exudation	Directly determines performance in P-limited organic soils	[49,50]
Soil carbon contribution	Root C:N ratio, rhizodeposition rate, aggregate-forming exudates	Root-trait-driven carbon sequestration is an overlooked ecosystem service of breeding value	[46,47]
Biodiversity-support traits	Floral resources, canopy architecture, rhizosphere chemistry shaping trophic networks	Genotype-dependent; links varietal choice to landscape-scale ecosystem services	[58,60]
Multi-stress physiological plasticity	Coordinated osmotic adjustment and rhizosphere signalling under combined drought/nutrient limitation	Genotypes selected for single-stress tolerance underperform under realistic combined-stress field conditions	[25,26]
Root exudate metabolomic profile	Composition (not quantity) of organic acids, flavonoids and mucilage determining recruited microbial guilds	Candidate marker trait for microbiome-informed marker-assisted selection	[43,62]

**Table 2:** Ecosystem services delivered by *Phaseolus vulgaris* L. and their genetic basis.

Service Category	Mechanistic Basis	Genotype-Dependence	Key References
Provisioning (food, feed, income)	Grain protein/micronutrient content; residue biomass	High; biofortification and residue quality are cultivar traits	[7,64]
Supporting (soil fertility)	BNF (45–110 kg N ha^−1^); P mobilization via root exudates	High; nodulation and exudation vary by genotype × strain	[50,67]
Regulating (carbon, pest, water)	Root biomass turnover; habitat complexity; aggregate-driven infiltration	Moderate-high; tied to root and canopy architecture	[59,60]
Cultural and agrobiodiversity	Landrace conservation; seed exchange networks	Intrinsic to landrace genetic diversity	[7,74]
Circular bioeconomy	Residue C:N ratio; decomposition and mineralisation rate	Moderate; residue quality is a selectable trait	[66,88]

**Table 3:** Ecosystem-oriented breeding targets derived from the principal agroecological limitations of *Phaseolus vulgaris* L.

Constraint	Mechanism	Breeding Target	Key References
Weed competition	Slow early canopy closure (0–45 DAE)	Early vigour/rapid canopy closure ideotype	[89,90]
Biotic stress (disease)	Rapid pathogen race evolution overcoming monogenic resistance	Durable, polygenic, microbiome-compatible resistance	[101,103]
Biotic stress (arthropods)	Direct feeding damage; virus transmission	Resistance compatible with conservation biocontrol	[108,112]
Nutrient limitation	P/K fixation in weathered soils constraining BNF	Nutrient-acquisition efficiency under low fertility	[116,120]
Combined abiotic stress	Drought + heat + flooding exceeding adaptive capacity	Multi-stress resilience (rooting depth, osmotic adjustment)	[126,127]
Organic seed/breeding gap	Conventional breeding ignores low-input trait space	Participatory, decentralized breeding for organic ideotypes	[135,138]

**Table 4:** Agroecological management practices and their dependence on host genetic background.

Practice	Primary Mechanism	Genotype-Dependent Component	Key References
Crop rotation	Disruption of host-specific pathogen cycles	Residue quality; disease-suppressive rhizosphere recruitment	[143,145]
Intercropping	Niche complementarity (light, water, nutrients)	Root architecture; canopy complementarity	[1,157]
Cover cropping	N supply and weed suppression via residue quality	Legume BNF capacity; residue C:N ratio	[166,167]
Organic fertilization	Slow-release nutrient cycling; biochar microsites	Nodulation response; nutrient-acquisition efficiency	[120,174]
Biostimulants/biopesticides	Induced stress tolerance; microbial antagonism	Overlaps with native rhizosphere-recruitment capacity	[183]

**Table 5:** Priority breeding research directions linking disciplinary knowledge gaps to selectable traits.

Discipline	Knowledge Gap	Priority Breeding Trait	Key References
Agronomy/soil ecology	Rotation legacy effects; root-carbon-driven aggregation poorly quantified	Residue quality; root-derived carbon input rate	[3,256]
Plant physiology	Mechanisms of P acquisition and C allocation under low input	Root plasticity; combined drought-heat ideotypes	[25,135]
Rhizosphere microbiology	Descriptive, not predictive, understanding of plant–microbe function	Microbiome-recruitment capacity as a heritable trait	[248]
Climate adaptation	Isolated- rather than combined-stress tolerance studied	Multi-stress (heat + drought) resilience	[269,270]
Digital agriculture	Algorithms uncalibrated for heterogeneous organic canopies	Traits compatible with UAV/sensor-based selection	[275,277]
Socio-economic/policy	Certification and seed-system barriers to diverse cultivars	Participatory-bred, seed-system-compatible ideotypes	[53,278]
Circular bioeconomy	Residue quality and mineralisation kinetics that are rarely bred for	Residue C:N ratio and decomposition rate as selectable output traits	[66,225]

## Data Availability

As this is a literature review, no new primary data were generated or analyzed in this study. All data discussed are derived from the publicly available sources cited in the reference list.

## Abbreviations

The following abbreviations are used in this manuscript:

AI	Artificial Intelligence
AMFs	Arbuscular Mycorrhizal Fungi
BNF	Biological Nitrogen Fixation
CRISPR	Clustered Regularly Interspaced Short Palindromic Repeats
DAE	Days After Emergence
EU	European Union
GWAS	Genome-Wide Association Study
IoT	Internet of Things
IPM	Integrated Pest Management
LER	Land Equivalent Ratio
NUE	Nitrogen-Use Efficiency
PGPRs	Plant-Growth-Promoting Rhizobacteria
QTL	Quantitative Trait Locus/Loci
SynComs	Synthetic Microbial Communities
UAV	Unmanned Aerial Vehicle
WUE	Water-Use Efficiency
